# Genome-scale metabolic reconstructions and theoretical investigation of methane conversion in *Methylomicrobium buryatense* strain 5G(B1)

**DOI:** 10.1186/s12934-015-0377-3

**Published:** 2015-11-25

**Authors:** Andrea de la Torre, Aisha Metivier, Frances Chu, Lieve M. L. Laurens, David A. C. Beck, Philip T. Pienkos, Mary E. Lidstrom, Marina G. Kalyuzhnaya

**Affiliations:** Biology Department, San Diego State University, North Life Science Room 406, San Diego, CA 92182-4614 USA; Viral Information Institute, San Diego State University, San Diego, USA; Department of Chemical Engineering, University of Washington, Seattle, USA; eScience Institute, University of Washington, Seattle, USA; Department of Microbiology, University of Washington, Seattle, USA; National Bioenergy Center, National Renewable Energy Laboratory, Golden, CO USA

**Keywords:** Methane metabolism, Flux balance model, *Methylomicrobium buryatense*, Strains 5G and 5G (B1), Metabolic engineering of methane utilization

## Abstract

**Background:**

Methane-utilizing bacteria (methanotrophs) are capable of growth on methane and are attractive systems for bio-catalysis. However, the application of natural methanotrophic strains to large-scale production of value-added chemicals/biofuels requires a number of physiological and genetic alterations. An accurate metabolic model coupled with flux balance analysis can provide a solid interpretative framework for experimental data analyses and integration.

**Results:**

A stoichiometric flux balance model of *Methylomicrobium buryatense* strain 5G(B1) was constructed and used for evaluating metabolic engineering strategies for biofuels and chemical production with a methanotrophic bacterium as the catalytic platform. The initial metabolic reconstruction was based on whole-genome predictions. Each metabolic step was manually verified, gapfilled, and modified in accordance with genome-wide expression data. The final model incorporates a total of 841 reactions (in 167 metabolic pathways). Of these, up to 400 reactions were recruited to produce 118 intracellular metabolites. The flux balance simulations suggest that only the transfer of electrons from methanol oxidation to methane oxidation steps can support measured growth and methane/oxygen consumption parameters, while the scenario employing NADH as a possible source of electrons for particulate methane monooxygenase cannot. *Direct coupling* between methane oxidation and methanol oxidation accounts for most of the membrane-associated methane monooxygenase activity. However the best fit to experimental results is achieved only after assuming that the efficiency of *direct coupling* depends on growth conditions and additional NADH input (about 0.1–0.2 mol of incremental NADH per one mol of methane oxidized). The additional input is proposed to cover loss of electrons through inefficiency and to sustain methane oxidation at perturbations or support uphill electron transfer. Finally, the model was used for testing the carbon conversion efficiency of different pathways for C_1_-utilization, including different variants of the ribulose monophosphate pathway and the serine cycle.

**Conclusion:**

We demonstrate that the metabolic model can provide an effective tool for predicting metabolic parameters for different nutrients and genetic perturbations, and as such, should be valuable for metabolic engineering of the central metabolism of *M. buryatense* strains.

**Electronic supplementary material:**

The online version of this article (doi:10.1186/s12934-015-0377-3) contains supplementary material, which is available to authorized users.

## Background

Methane, as a pipeline-based, cheap source of carbon is becoming an attractive feedstock for biosynthesis [1–4]. Taking into account that many human-generated sources of methane represent “hot spots” of greenhouse gas emissions, including landfills, wastewater treatment plants, and manure management facilities, the biological conversion of methane represents a novel and potentially transformative solution for a number of environmental challenges associated with urbanization and industrial growth [5–7].

Methane is a natural element of the global carbon cycle [8, 9]. The majority of methane produced in nature is consumed by microbes [9, 10]. A number of microbial phyla are capable of methane conversion [4, 10, 11]; however only a subset of microbes displays characteristics that could be appropriate for industrial applications [4, 10]. Furthermore, the application of natural methanotrophic strains to large-scale production of value-added chemicals/biofuels requires a number of physiological and genetic alterations [1, 2, 4, 10].

In the last decade, metabolic models of microorganisms have gone from a small set of reductionist models to whole-(meta)genome models of wide variety of organisms and consortia. Metabolic modeling became a useful tool for in silico experiments with whole-cell metabolic phenotyping and engineering [12, 13]. While the genomics and biochemistry of microbial C_1_-metabolism are relatively well established, only a few mathematical descriptions of methane or methanol utilization have been developed [14–16]. It has been assumed that growth of C_1_-compounds is reducing power limited [16, 17]. The prediction is well supported by metabolic modeling of methanol utilization [15]. Contrary to methylotrophy models, the theoretical calculation of methanotrophy has shown very poor correlation with measured parameters [18, 19]. Incorrect assumptions regarding the core metabolic arrangements of methane oxidation and/or assimilation could account for this discrepancy. No validated whole genome-scale metabolic model (GSM) of a methane-utilizing microbe has yet been published. However, access to the complete genome sequences of methanotrophic bacteria has now provided new top-down approaches for initial metabolic reconstruction [13, 20, 21, 22]. A number of genome-scale biochemical network reconstructions of biotechnology-relevant methanotrophic bacteria are available in BioCyc (http://www.biocyc.org). However, these are mostly based on automatic annotation pipelines, which commonly do not recognize pathways associated with single carbon utilization. In this work we present a stoichiometric metabolic model of *Methylomicrobium buryatense* 5G(B1) [23–26]. The genome-based reconstruction was further validated by comparison of model predictions to physiological measurements. The model was used to evaluate different metabolic arrangements of methane oxidation and assimilation. The metabolic model of methane oxidation that most accurately simulates the interplay between experimental measurements (methane consumption rate) and performance of the biological system (growth rate, substrate consumption and biomass yield) was further used to calculate the most efficient pathways for biomass production with different sources of nitrogen and sulfur as growth nutrients.

## Results and discussion

### Metabolic network reconstruction

In this study we used the genome sequence of *Methylomicrobium buryatense* strain 5G [27]. The metabolic network of strain 5G is interchangeable with that of strain 5GB1, a derivative of strain 5G [25]. To mathematically model the methane utilization network we used *PathwayTools™* (http://bioinformatics.ai.sri.com/ptools/). This bioinformatics platform provides a one-point solution for the development, integration, and visualization of multi-scale heterologous systems biology data, including comparative analyses of organism-specific databases, reconstruction of metabolic pathways/networks, execution and curation of steady-state metabolic flux models, phenotypic predictions, and metabolic engineering.

The genome-scale metabolic network reconstruction was based on the whole genome sequence of wild type *Methylomicrobium buryatense* strain 5G (GenBank/EMBL under the accession numbers AOTL01000000 and KB455575 and KB455576) [27]. The complete list of genes was downloaded from the IMG (JGI) website and imported into *PathwayTools™* (http://bioinformatics.ai.sri.com/ptools/) as described in “Methods”.

The initial GSM contained metabolic reactions that were predicted based on automated genome annotation. Gene Ontology (GO) terms were added in addition to the Enzyme Commission (EC) assignments for improved automated model construction with PathoLogic. The PathoLogic test parsing was performed and any missing enzymes and holes were flagged for further manual inspection (approximately 560 reactions/holes). Additional manual curating of the annotations was required, as automatic annotation does not correctly predict most methanotrophic pathways. The existing annotations were updated against an expert-curated database of methanotroph genomes (OMeGA, genomes uploaded at https://www.genoscope.cns.fr). Initial reconstruction included 1455 reactions arranged in 267 pathways. More than 1/3 of the automatically predicted reactions were removed and 100 new reactions (40 new pathways) were added. The final model, named *i*Mb5G(B1), includes 841 enzymatic reactions, arranged in 167 metabolic pathways. The list of reactions is presented in Additional file 1: Table S1.

### Metabolic network overview

**Fig. 1:**
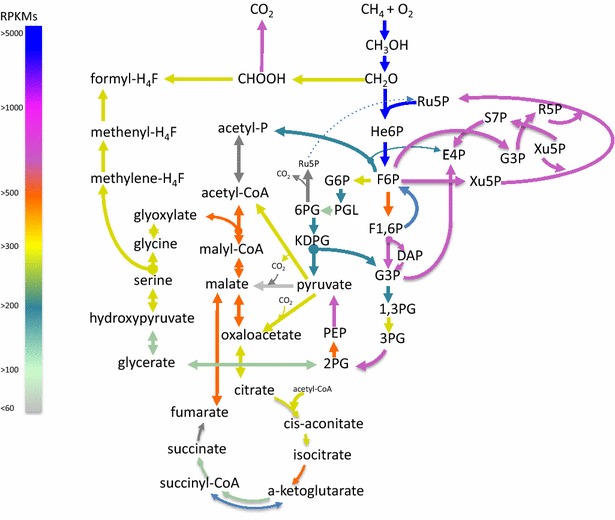
Overview of central metabolic pathways in *M. buryatense* 5GB1 predicted from genomic and transcriptomic data; *Color* indicates level of relative gene expression: very *high* (>5000RPKM); *high* (>1000); *intermediate* (>500), *low* (>200), *very low* (>100) and “*not expressed*” (<60 RPKM, shown in *grey*). *Ru5P* ribulose 5-phosphate, *He6P* 3-hexulose 6-phosphate, *F6P* fructose 6-phosphate, *KDPG* 2-keto-3-deoxy 6-phosphogluconate, *F1,6P* fructose 1,6-bisphosphate, *DAP* dihydroxyacetone phosphate, *G3P* glyceraldehyde 3-phosphate, *3PG* 3-phosphoglycerate, *2PG* 2-phosphoglycerate, *PEP* phosphoenolpyruvate, *6PG* 6-phosphogluconate, *S7P* sedoheptulose 7-phosphate, *E4P* erythrose 4-phosphate, *R5P* ribose 5-phosphate, *X5P* xylulose 5-phosphate, *G6P* glucose 6-phosphate, *G1,3P* glycerate-1,3-bisphosphate

**Fig. 2:**
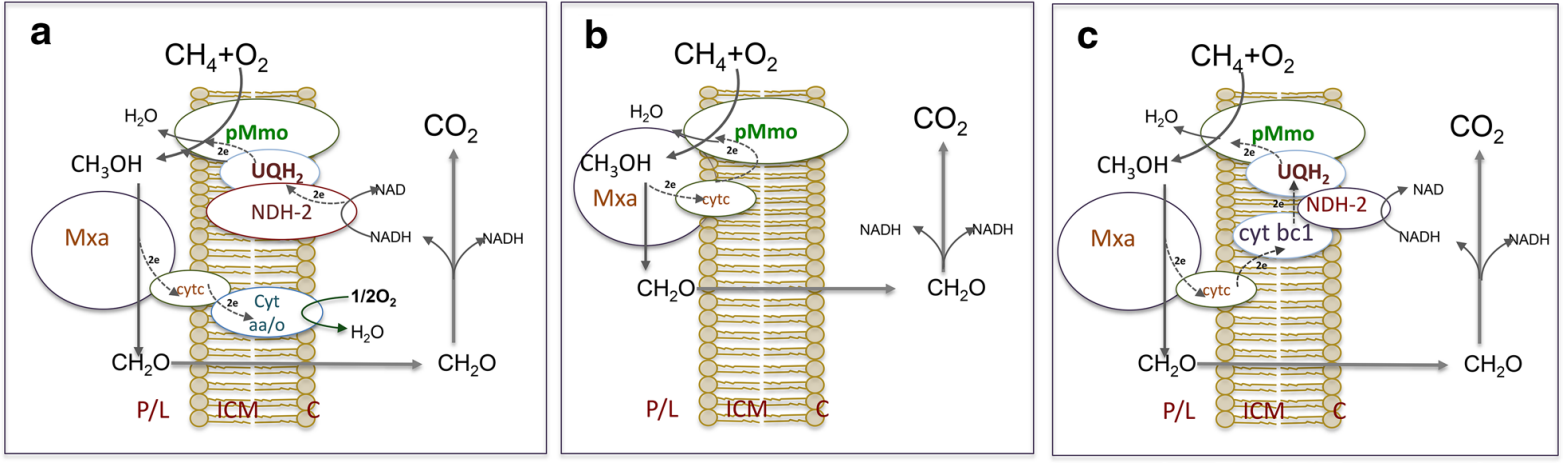
Possible modes of methane oxidation for methane-grown *M. buryatense* 5GB1. *Upper panel* a genome scale FBA was used to test three possible modes of methane oxidation: **a** *redox*-*arm mode*, the currently accepted model in which electrons driving methane oxidation come from NADH produced by formate or formaldehyde oxidation, while electrons produced from methanol oxidation are linked to a redox-arm and used for ATP production (adapted from Semrau et al. [30]); **b** *direct coupling mode*, in which methanol oxidation supplies electrons for methane oxidation; and **c** *uphill electron transfer* model, in which methanol oxidation partially supports methane oxidation (from [18]). Proton translocation and ATP synthesis were omitted for simplicity. *P* periplasm, *L* ICM (lumen), *C* cytoplasm

A summary of methane metabolism in *M. buryatense* is shown in Fig. 1. Methane oxidation in *M. buryatense* 5G(B1) can be driven by either of two enzymatic systems: membrane-associated methane monooxygenase (pMMO) or soluble methane monooxygenase (sMMO). sMMO uses NADH as a source of reducing power. The exact nature of the electron source for the pMMO is not known. It has been proposed that endogenenous ubiquinol (QH_2_), reduced by a membrane-associated formaldehyde and/or formate dehydrogenases or a type 2-NADH:quinone oxidoreductase (NDH-2) is the in vivo electron donor [28, 29]. *Uphill electron transfer* has also been proposed to explain the relatively high carbon conversion efficiencies observed in methanotrophic bacteria [19]. An alternative to *uphill electron transfer* is direct transfer of electrons (*direct coupling*) from methanol dehydrogenase (MDH) to pMMO [19, 30, 31]. In our initial model, the electron source for the pMMO is represented as a “reduced electron acceptor”. The product of the reaction, an “oxidized electron acceptor”, was projected to be reduced in one of three ways: (1) via the NADH:quinone oxidoreductase complex to incorporate the currently accepted *redox arm* model, in which electrons from methanol oxidation support ATP production while formate and/or formaldehyde oxidation support methane oxidation [29]; (2) via MDH, to represent a *direct coupling model* [30, 31]; or (3) via MDH and *uphill electron transfer* supported by NADH [19]. All three arrangements of methane oxidation machinery are shown in Fig. 2. Each arrangement of the electron acceptor reduction was independently modeled and outcomes of the initial step of methane oxidation are described and discussed below.

The second step of the methane oxidation pathway is catalyzed by a periplasmic pyrroloquinoline quinone (PQQ)-linked MDH [23, 24]. Two systems could contribute to observed enzymatic activity: a two-subunit Mxa system [32], and a monosubunit Xox-enzyme [33, 34]. Genes encoding both enzymes are present in the genome of *M. buryatense* strain 5G [27].

Genome annotation predicts multiple pathways for formaldehyde oxidation, including (1) tetrahydromethanopterin- and (2) tetrahydrofolate-linked C_1_-transfer pathways, (3) formaldehyde dehydrogenase and (4) oxidative pentose phosphate cycle (oxPPP, also known as oxidative ribulose monophosphate cycle). Two enzymatic systems, both NAD-linked formate dehydrogenases, are predicted for formate oxidation.

Three variants of the ribulose monophosphate, the Embden-Meyerhof-Parnas pathway (EMP), the Entner–Doudoroff (EDD) pathway [27] and Bifidobacterial shunt (BS) [But and Kalyuzhnaya, unpublished], are predicted based on the gene inventory. All three pathways are interconnected with the transaldolase variant of the pentose phosphate pathway for regeneration of ribulose-5-phosphate, a metabolic acceptor for formaldehyde. All three pathways were manually reconstructed and linked with corresponding genes.

The genome of *M. buryatense* 5G encodes all genes essential for operation of the citric acid cycle (TCA) and most of the genes for the serine cycle. However, no homologs of the phosphoenolpyruvate carboxylase, glyoxylate shunt, or the ethylmalonyl-CoA pathway for glyoxylate regeneration enzymes were found. Thus, the strain cannot use the serine cycle as a sole pathway for C_1_-assimilation [4, 11]; however, a partial serine cycle might still contribute to carbon conversion to acetyl-CoA (Fig. 1). The partial serine cycle must include a pyruvate carboxylase and malate dehydrogenase or malic enzyme for malate production.

The details for each of the pathways identified downstream of core metabolism were verified manually. Biosynthetic pathways for fatty acid, sucrose, nucleic acid, amino acid, ubiquinol-8, tetrahydrofolate, cell wall components (LPS and murein), cofactors and vitamins, squalene and lanosterol, as well as nitrogen fixation and sulfate, nitrate, ammonia, urea and phosphate utilization were manually corrected or reconstructed. Each reaction was tested for mass balance and irreversibility constraints. The model was further tested for accuracy by analyzing gene expression data.

### Initial model refinement: transcriptomic data

RNA samples extracted from *M. buryatense* 5GB1 grown on methane to exponential phase were sequenced using the Illumina^®^ platform. The replicates were aligned to the reference genome using BWA under default parameters (see “Methods”). Transcriptomic data (Fig. 1a; Additional file 2: Table S2) were used to refine core metabolic pathways and amino acid, fatty acid, phospholipid and cofactor biosyntheses. Furthermore, the following assumptions were incorporated into the FBA analysis based on the transcriptomic data: (1) Unless tested, the flux through the NADH-dependent methane oxidation reaction was fixed at 0, as no expression of soluble methane monooxygenase is observed at tested growth conditions (Cu 9 μM); (2) Methanol oxidation occurs only in the periplasm; a housekeeping alcohol dehydrogenase 2 (*ald2*) was set to contribute to detoxification of small amounts of C_1_-alcohol produced in biosynthetic reactions in the cytosol; (3) The model allows free inter-conversion between NADH and NADPH, as NAD(P)-transhydrogenase was found to be expressed; (4) The flux through the malic enzyme was fixed at 0, as no expression of the gene was observed.

### Biomass composition

The biomass composition is summarized in Table 1. The following compounds were measured in this study: amino acids, fatty acids, phospholipid composition, squalene and lanosterol, exopolysaccharides (EPS), lipopolysaccharides (LPS), and nucleic acids (DNA and RNA). All parameters were measured in exponentially grown cells of *M. buryatense* 5GB1 (see “Methods”). The small molecule composition of exponentially grown cells is almost identical to that measured for unlimited, fed-batch growth in a bioreactor [26]. The rest of the small molecule cell composition was compiled upon examination of primary literature [23, 24, 35, 36, 37, 38, 39, 40, 41, 42]. Intracellular concentrations of cofactors and vitamins were assumed to be present in similar proportions to *M. alcaliphilum* 20z [36, 37, 40] or *E. coli* [38, 39]. Intracellular concentrations of carbohydrates (mannose, ramnose, glucose, galactose, ribose, maltose, cellulose, sucrose, arabinose), phosphosugars (fructose-6-phosphate, fructose-bisphosphate, glucose-6-phosphate, 6-phosphogluconate), phosphoenolpyruvate, acetyl-CoA, and organic acids (malate, citrate, pyruvate, succinate) were assumed to be similar to *M. alcaliphilum* 20z [24, 41]. Glycogen as a polymer of undefined length was converted to equivalent quantities of glucose [26]. The fatty acid profile of *M. buryatense* strain 5GB1 is dominated by hexadecanoic (C_16:0_) and hexadecanoic acid (C_16:1_) isomers. The C_16_-fatty acid isomers are represented in the model as palmitate.

**Table 1:** The biomass composition and general growth parameters of the *M. buryatense* 5G(B1) cells

Compound	%	SD	mmol/g DCW biomass	Organism source	References
Amino acids	54.8	3.1		*M. buryatense* 5GB1	
Alanine			0.446		This study
Arginine			0.202		This study
Asparagine			0.119		This study
Aspartate			0.348		This study
Cysteine			0.03		This study
Glutamate			0.396		This study
Glutamine			0.15		This study
Histidine			0.088		This study
Glycine			0.443		This study
Isoleucine			0.256		This study
Leucine			0.26		This study
Lysine			0.25		This study
Methionine			0.12		This study
Phenylalanine			0.196		This study
Proline			0.195		This study
Serine			0.23		This study
Threonine			0.26		This study
Tryptophan			0.072		This study
Tyrosine			0.14		This study
Valine			0.341		This study
Ectoine	0.38	0.1	0.027	*M. buryatense* 5G	[23, 24]
Lipids					
FAME	10.9	0.56		*M. buryatense* 5GB1	This study
C14 (myristate)			0.016		This study
C15 (pentadecylic acid)			0.002		This study
C16 (palmitate)			0.307		This study
C18 (stearate)			0.001		This study
Phospholipids				*M. buryatense* 5G	Recalculated from [23]
Phosphatidylserine*			0.014		[23]
Phosphatidylethanolamine*			0.122		[23]
Dipalmitoyl phosphatidate*			0.007		[23]
Phosphatidylglycerol*			0.031		[23]
Sterols	0.08	–	0.08	*M. buryatense* 5GB1	This study
squalene			0.04		
lanosterol			0.04		
Intracellular metabolites	1.5	–			
Ribulose-5-phosphate/ribose-5-phosphate			0.001	*M. alcaliphilum* 20Z	[42]
Fructose-1, 6-bisphosphate			0.001	*M. alcaliphilum* 20Z	[42]
Fructose-6-phosphate			0.003	*M. alcaliphilum* 20Z	[42]
Glucose-6-phosphate			0.002	*M. alcaliphilum* 20Z	[42]
Glyceraldehyde-3-phosphate/dihydroxyacetone			0.003	*M. alcaliphilum* 20Z	[42]
6-Phosphogluconic acid			0.00015	*M. alcaliphilum* 20Z	[42]
2-Dehydro-3-deoxy-phosphogluconate			0.000003	*M. alcaliphilum* 20Z	[42]
Phosphoglycerate			0.006	*M. alcaliphilum* 20Z	[42]
Phosphoenolpyruvate			0.005	*M. alcaliphilum* 20Z	[42]
Pyruvate			0.015	*M. alcaliphilum* 20Z	[42]
Acetyl-CoA			0.0001	*M. alcaliphilum* 20Z	[42]
Succinate			0.002	*M. alcaliphilum* 20Z	[42]
Malate			0.004	*M. alcaliphilum* 20Z	[42]
Fumarate			0.001	*M. alcaliphilum* 20Z	[42]
Citrate			0.001	*M. alcaliphilum* 20Z	[42]
Glycerate			0.001	*M. alcaliphilum* 20Z	[42]
ATP			0.005	*Methylomonas methanica*	[43, 44]
ADP			0.002	*M. extorquens* AM1	[16]
AMP			0.001	*M. extorquens* AM1	[16]
NAD			0.002	*M. extorquens* AM1	[16]
NADH			0.002	*M. extorquens* AM1	[16]
NADP			0.001	*M. extorquens* AM1	[16]
NADPH			0.001	*M. extorquens* AM1	[16]
polyP (PPi)			0.029	*Methylomonas methanica*	[43, 44]
Cofactors					
Cytochrome c			0.00036	*M. alcaliphilum* 20Z	[37]
B12			0.00000006	*M. alcaliphilum* 20Z	[41]
Ubiquinol-8			0.00022	Assumption	[16]
Protoheme			0.00022	Assumption	[16]
coenzyme-A			0.00022	Assumption	[16]
FMN			0.00022	Assumption	[16]
FMNH2			0.00022	Assumption	[16]
FAD			0.00022	Assumption	[16]
SAM			0.00022	Assumption	[16]
Glutathione			0.00022	Assumption	[16]
Carbohydrates	3.78	1.56			
Mannose			0.002	*M. alcaliphilum* 20Z	[24]
Ramnose			0.0002	*M. alcaliphilum* 20Z	[24]
Glycogen			0.117	*M. buryatense* 5GB1	[26]
Sucrose			0.01	*M. alcaliphilum* 20Z	[45]
Ribose			0.036	*M. alcaliphilum* 20Z	[24]
Maltose			0.008	*M. alcaliphilum* 20Z	[24]
Arabinose			0.022	*M. alcaliphilum* 20Z	[24]
Galactose			0.005	*M. alcaliphilum* 20Z	[24]
Cell wall	9.127				
Peptidoglycan		–	0.053	*Escherichia coli*	[46]
LPS (lipid IVA and KDO)		–	0.002	*M. buryatense* 5GB1	This study
RNA	9.7	3.6		*M. buryatense* 5GB1	This study
ATP			0.050		
UTP			0.050		
CTP			0.047		
GTP			0.047		
DNA	4.0	0.28		*M. buryatense* 5GB1	This study
dATP			0.0021		
dTTP			0.0021		
dCTP			0.0020		
dGTP			0.0020		
Ash (without P and S data)	5.2	1.03	–	*M. buryatense* 5GB1	[27]
Copper			0.01		
Magnesium			0.07		
Iron			0.0059		
Cobalt			0.001		
Calcium			0.01		
Biomass (measured)	99.5	10.2			
3-PG	0.5		0.003		
Total	100.0				
Excreted products					
Formate	2.8	0.17	0.6	*M. buryatense* 5GB1	[27]
Acetate	0.7	0.02	0.114	*M. buryatense* 5GB1	[27]
Lactate	0.4	0.13	0.044	*M. buryatense* 5GB1	[27]
EPS	10	4.5		*M. buryatense* 5GB1	This study
Glucose			0.0420	*M. buryatense* 5GB1	This study
Fucose			0.0075	*M. buryatense* 5GB1	This study
Xylose			0.0023	*M. buryatense* 5GB1	This study
Inositol			0.0021	*M. buryatense* 5GB1	This study
Galactose			0.0588	*M. buryatense* 5GB1	This study
Mannose			0.0669	*M. buryatense* 5GB1	This study
Ribose			0.0342	*M. buryatense* 5GB1	This study
Rhamnose			0.0432	*M. buryatense* 5GB1	This study
Glucosamine			0.0415	*M. buryatense* 5GB1	This study
Galactosamine			0.0394	*M. buryatense* 5GB1	This study
Cysteine			0.0185	*M. buryatense* 5GB1	This study
Threonine			0.0375	*M. buryatense* 5GB1	This study
Serine			0.0214	*M. buryatense* 5GB1	This study
Glutamate			0.0319	*M. buryatense* 5GB1	This study
Glycine			0.0830	*M. buryatense* 5GB1	This study
Valine			0.0228	*M. buryatense* 5GB1	This study
Methionine			0.0066	*M. buryatense* 5GB1	This study
Isoleucine			0.0146	*M. buryatense* 5GB1	This study
Leucine			0.0169	*M. buryatense* 5GB1	This study
Phenylalanine			0.0139	*M. buryatense* 5GB1	This study
Growth parameters					
Methane uptake (mmol g CDW^−1^ h^−1^)			18.46 ± 1.36	*M. buryatense 5GB1*	[27]
Oxygen uptake (mmol g CDW^−1^ h^−1^)			23.55 ± 1.13	*M. buryatense 5GB1*	[27]
O_2_/CH_4_ uptake Ratio			1.25 ± 0.05	*M. buryatense 5GB1*	[27]
Specific growth rate (h^−1^)			0.232 ± 0.006	*M. buryatense 5GB1*	[27]

### Input parameters and main energetic assumptions

The validity of this model in application to methanotrophic metabolism was assessed by comparing the theoretical predictions with experimental results. We ran a set of in silico flux balance trials using the software to validate the stoichiometric completeness of the proposed metabolic network. The experimental parameters outlined below were used for FBA simulations. The nutrient composition was based on the typical composition of growth medium: methane as a source of carbon; nitrate, sulfate and phosphate as sources of N, S and P, respectively. Additional constraints were added as follows: (1) Only water and CO_2_ are included as the expected excretion products. However, the growth of *M. buryatense* strain 5GB1 is accompanied by accumulation of formate (0.6 mmol g^−1^ DCW), acetate (0.1 mmol g^−1^ DCW), lactate (0.044 mmol g^−1^ DCW) [26] and extracellular polymeric substances (EPS) (0.1 g g^−1^ DCW). These organic compounds were also incorporated as part of the biomass equation and included in the biomass flux; however, they were deducted from biomass growth; (2) methane consumption rate was set at 18.46 mmol g^−1^ DCW h^−1^, based on measurements taken during unlimited growth [26].

All reactions involved in methane, methanol, and formaldehyde oxidation (except for the tetrahydrofolate pathway reactions) were set as irreversible. It has been demonstrated that in methylotrophs, methanol oxidation operates as a *redox arm* and is coupled with ATP generation with 0.5–1 mol of ATP produced per 1 mol of methanol oxidized (Fig. 2a) [45]. Both parameters (0.5 and 1 mol of ATP per 1 mol methanol) were used to test the *redox arm* model. For *uphill electron transfer* and *direct coupling*, this parameter was set to 0, as in these scenarios it is assumed that electrons from methanol oxidation are used for methane oxidation (Fig. 2b, c).

Several assumptions were made regarding electron transport and oxidative phosphorylation. Since the majority of electron transfer reactions are not yet known in methanotrophic bacteria, it is assumed that the H+/ATP ratio, or the number of protons translocated per one ATP synthesized during respiration, was 3.3 [47]. Based on the genome sequence, it could be predicted that the electron transfer chain in the methanotroph includes complex I, complex III and complex IV, with an expected yield of H^+^/NADH = 8.32 (with 92 % efficiency) [47–49]. Thus oxidation of 2 NADH could result in the production of 6 ATP. The complex set of electron transport and oxidative phosphorylation reactions in the model is described by one equation:

$$\begin{aligned} &2 {\text{NADH}}/{\text{H}}^{ + } + {\text{ O}}_{ 2} + {\text{ 6ADP }} + 5 {\text{Pi}} \\& = {\text{ 2NAD }} + {\text{ 7H}}_{ 2} {\text{O }} + {\text{ 6ATP}}\end{aligned}$$

Additional tests with low ATP yield (2.7 and 2.5 mol of ATP per 1 mol NADH) were also performed. A membrane-bound, proton-translocating pyrophosphatase plays an important role in methanotrophic metabolism, as it supports the activity of pyrophosphate-dependent phosphofructokinase, a core enzyme of the EMP pathway, by supplying pyrophosphate (PPi). The predicted ratio of ATP hydrolysis:PPi formation for this class of enzymes is 1:3 [50]. Non-growth-associated ATP maintenance was set to 8.39 mmol ATP g^−1^ CDW h^−1^ [51–54]. Since growth-associated ATP maintenance could vary depending on the growth rate of cells [52, 53], two settings were tested: ATP maintenance with the cost being set at 23 mmol ATP g^−1^ CDW (e.g., the same as a low value used for *E. coli* [52]) is shown as “*low ATP maintenance*” in the output files; and ATP maintenance being set at 59.81 mmol ATP g^−1^ CDW (e.g., the same as a high value used for *E. coli* [54].

Unless specifically tested, it was assumed that 75 % of the intracellular pyruvate is produced via the EMP pathway and 25 % of the intracellular pyruvate comes from the EDD pathway, the same as in *Methylomicrobiumalcaliphilum* 20z, as the gene expression profile and corresponding enzyme activities are similar to those in *M.alcaliphilum* 20z [55, 42]. A number of ABC-transporters for nitrate, sulfate, sulfide, ammonium, iron, copper, magnesium and phosphate transport have been identified in the genome sequence. All transport reactions are listed in Additional file 3: Table S3. A set of transport reactions for subcellular relocation of C_1_-compounds (from the extracellular space to the periplasm and then to the cytosol) were introduced into the model. However, since no specific system for C_1_-transport is known or could be predicted from gene expression data, it was assumed that methane, methanol or formaldehyde transport reactions do not require any additional energy input.

### Modeling *M. buryatense* 5GB1 growth on methane: *redox arm* vs *direct coupling* vs *uphill electrons*

The model recruited up to 402 reactions to synthesize all 118 metabolites, which were included in the biomass composition. The model was used to test the three possible modes of methane oxidation: the *redox*-*arm mode*, the currently accepted model in which electrons driving methane oxidation come from NADH produced by formate or formaldehyde oxidation [29], while electrons produced from methanol oxidation are linked to the *redox*-*arm* and used for ATP production (Fig. 2a); the *direct coupling mode*, in which methanol oxidation supplies electrons for methane oxidation without any additional inputs (Fig. 2b); and *uphill electron transfer*, in which electrons from the methanol oxidation step go uphill to support methane oxidation (Fig. 2c), and the *uphill electron transfer* is supported by additional input from complex I and complex III.

The output parameters for each mode, the predicted growth rate of the strain, the carbon conversion efficiency (CCE) and O_2_:CH_4_ consumption ratios were compared to experimental measurements (Table 1) [26]. Briefly, the maximum growth rate of the strain is 0.232 h^−1^ and the O_2_:CH_4_ consumption ratio at the maximum growth is 1.25 ± 0.05. The consumption ratios and the measured biomass growth and yield (represented as CCE) vary depending on growth conditions [26]. The lowest biomass yield was observed during oxygen-limited growth (CCE = 44.5 %, at O_2_:CH_4_ consumption ratio of 1.15) and the highest yield was observed during methane limited growth (CCE = 57.5 % at O_2_:CH_4_ consumption ratio of 1.6).

**Fig. 3:**
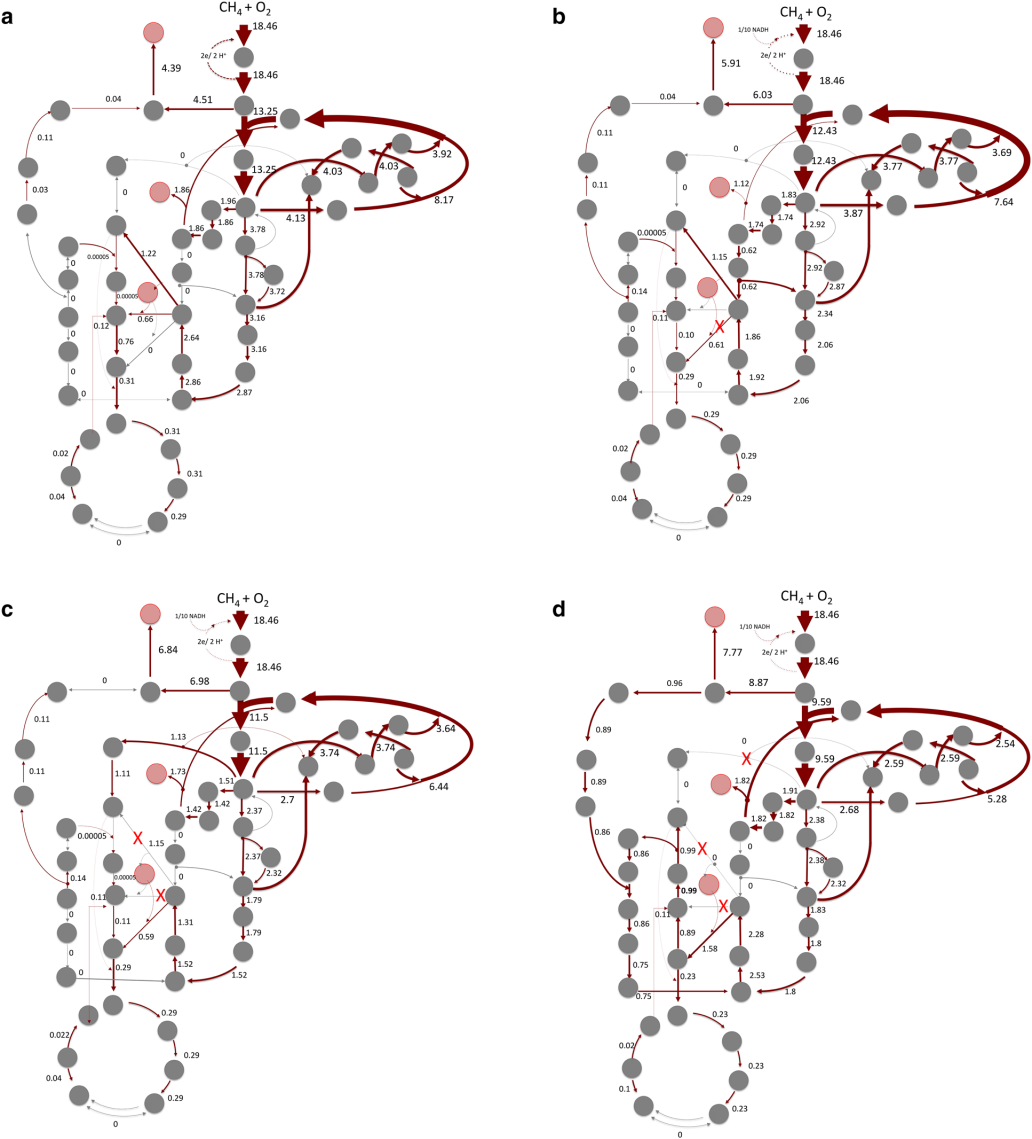
Carbon flux distributions in *M. buryatense* 5GB1. **a** Unconstrained network; **b** application of known carbon flux distribution between EMP and EDD pathways [41]; **c** network predicted for a pyruvate dehydrogenase mutant; **d** network predicted for a pyruvate dehydrogenase mutant and phosphoketolase mutant. Acetyl-CoA is produced via partial serine cycle. Steps with no flux are shown in *grey*

The unconstrained network selected the *direct coupling* as the most optimal solution for methane oxidation (Fig. 3a). The *direct coupling* mode predictions for growth rate and CCE correlated well with the experimental data for biomass flux (Table 2). The predicted O_2_:CH_4_ ratio of 1.16 is at the lowest end of the measured parameters [26]. However, the biomass yield is higher than measured. Direct coupling linked to low ATP-yield reduced the biomass flux and increased cell requirements for oxygen. At this setting, the outputs of the model were close to experimental data, suggesting that the *direct coupling mode* of methane oxidation is one of the possible modes of methane oxidation when it co-occurs with active respiration. An alternative explanation could be that the *direct coupling* is not efficient, co-occurring with some spontaneous loss of electrons (such as spontaneous coupling with oxygen or coupling with cytochrome c oxidase). In that case, the loss of electrons would be refilled by Complex I/III. To evaluate this hypothesis, we tested two scenarios in which a part of the NADH produced from formaldehyde oxidation was used to replenish (reenergize) methane oxidation. The outcomes of the scenario 1 (1 mol of NADH supports oxidation of 10 mol of methane) correlate best with all experimental data obtained for unlimited growth on methane (Table 2).

**Table 2:** Computation predictions for different modes of methane oxidation and growth conditions

	Biomass flux^a^	Oxygen consumption	O_2_:CH_4_ ratio	CO_2_ production	CCE
Unconstrained network	0.246	21.34	1.16	7.78	57.85
Methane oxidation mode					
Direct coupling	0.242	21.59	1.17	7.95	56.93
Direct coupling/low ATP maintenance	0.263	20.24	1.10	7.02	61.97
Direct coupling/low ATP yield	0.238	21.82	1.18	8.1	56.12
Low efficiency direct coupling, complemented by NADH (1 mol of NADH per 10 mol of methane oxidized)^b^	0.23	21.43	1.16	8.47	54.12
Low efficiency direct coupling, complemented by NADH (1 mol of NADH per 5 mol of methane oxidized)	0.219	21.2	1.15	8.94	51.57
Uphill electron transfer/ NADH input (2/3 electrons from cytochrome cL and 1/3 from NADH)	0.224	21.8	1.18	8.72	52.76
Uphill electron transfer/NADH input (1/2 electrons from cytochrome cL and 1/2 from NADH)	0.205	22.99	1.25	9.53	48.37
Redox arm (0.5 mol ATP per 1 mol methanol oxidized)	0.146	27.69	1.50	12.13	34.29
Redox arm (1 mol ATP per 1 mol of methanol oxidized)	0.146	27.69	1.50	12.13	34.29
Redox arm (1 mol ATP per 1 mol of methanol oxidized)/low ATP maintenance	0.146	27.69	1.50	12.13	34.29
Redox arm (0.5 mol ATP per 1 mol methanol oxidized)/high ATP maintenance	0.142	27.92	1.51	12.29	33.42
Assimilation pathways (with low efficiency direct coupling complemented by NADH)^a^					
EMP only	0.232	21.28	1.15	8.37	54.66
EDD only	0.223	21.85	1.18	8.76	52.55
BS (acetyl-CoA synthesis only)	0.228	21.55	1.17	8.56	53.63
Serine cycle (acetyl-CoA synthesis only)	0.222	21.93	1.19	8.82	52.22
Nitrogen source					
Nitrogen fixation	0.228	23.23	1.26	8.58	53.52
Ammonium	0.29	22.08	1.20	5.88	68.15
Urea^c^	0.293	21.93	1.19	6.86	62.84
Sulfur source					
HS^−^	0.233	21.35	1.16	8.37	54.66
HS^−^ and Urea^c^	0.295	21.93	1.19	6.79	63.22

^a^Methane uptake is 18.46 mmol g CDW ^−1^ h^−1^

^b^Methane oxidation mode used to calculate data shown here for different sources of nitrogen and sulfur

^c^Only CO_2_ from methane was used to calculate CCE

The *redox*-*arm* model predictions were significantly above the measured range for O_2_:CH_4_ ratios (1.5), while the growth rate and CCE were too low (Table 2). The output parameters of this mode did not change at low-ATP maintenance and very high ATP-yield (one mol ATP per mol of methanol oxidized), suggesting that the system is reducing power limited rather than energy limited. In that mode, none of the NADH produced from formaldehyde oxidation was used in respiration. Furthermore, at settings described only at high ATP maintenance and low ATP-yield, the flux via a futile cycle for ATP oxidation approached zero. In summary, the *redox*-*arm* model of methane oxidation is not supported by the experimental results, suggesting the need for a metabolic shunt for electrons from methanol oxidation to fulfill the energetic needs of the first step of oxidation.

*Uphill electron transfer* has been suggested as an alternate mode, which might account for lower measured yields and oxygen consumption. The exact input of energy needed to drive *uphill electron transfer* has never been measured. Here, we tested two additional scenarios: (1) with 1/3 NADH input, while electrons from methanol oxidation enter the respiration chain (2 ATP is produced per 10 mol of methane converted to formaldehyde); and (2) 1/2 of NADH input, while electrons from methanol oxidation enter the respiration chain (2 ATP is produced per 10 mol of methane converted to formaldehyde). Predictions from scenario 1 show some correlation with biomass growth, while scenario 2 shows good correlation with oxygen-consumption at unlimited conditions.

Based on our in silico simulation data and experimental parameters, we propose that methane oxidation should be considered as a dynamic process that depends on electron transfer from a methanol oxidation step to methane oxidation. The exact nature of the transfer remains to be established. It should be kept in mind that for all in silico experiments the only fundamental difference between low-efficiency *direct coupling* and *uphill electron transfer* is the amount of NADH needed to drive methane oxidation; the exact organization of the oxidation machinery still requires additional experimental validation. In addition, it is possible that some other part of the metabolic network is not correctly reconstructed. However, the triple constraints of growth rate, CCE, and O_2_;CH_4_ ratio and the body of evidence supporting the components of the metabolic network make it unlikely that an error in the metabolic network could be sufficiently large to support the *redox arm* results.

Confirmation of this part of C_1_-biocatalysis awaits additional experimental data. However, a *direct coupling* with an input from respiration was assumed to be the most likely mode for methane oxidation, since it correlates best with the experimental results. That mode has been used to test the contribution of different assimilatory pathways.

### Methane assimilation

Figure 3a shows the unconstrained flux distribution for core metabolic pathways of *M. buryatense* 5G(B1) for the *direct coupling* metabolic mode. The flux distribution between the EMP and EDD pathways was set at 3 to 1 as previously reported for a related strain (Fig. 3b) [42]. Unless this parameter was set, the model does not predict any carbon flux via the EDD-pathway. When the carbon flux via the EMP pathway is set for 0, the predicted growth rate and CCE are slightly reduced and the O_2_:CH_4_ consumption ratio is slightly increased (Table 2). This result could be predicted based on the overall balance differences between EMP and EDD pathways. When all carbon is predicted to be assimilated via the EMP pathway, the growth rate and CCE are slightly increased. These differences have relatively minor impacts on the predicted parameters.

It has been predicted that pyruvate (the main product of the EMP and EDD pathways) is the main precursor for acetyl-CoA production [56]. Recent genomic studies suggest that methanotrophic bacteria might possess an additional variant of the RuMP pathway that can result in the production of C_2_ compounds via a phosphoketolase (Xfp) step (But and Kalyuzhnaya, unpublished). The incorporation of phosphoketolase is predicated to significantly improve the carbon conversion efficiencies of glucose-utilizing microbes, as well as methanol-consuming, engineered microbes [57, 58]. Here we tested if incorporation of Xfp might impact CCE in methane-utilizing microbes. The FBA does not require Xfp as an optimal flux when a pyruvate dehydrogenase (pdh) step is present. In order to require flux via an Xfp step, we ran a simulation experiment with an in silico **Δ***pdh* knock-out (Fig. 3c). In that case, all acetyl-CoA was produced via the Xfp step, but the overall CCE dropped as more C_1_-carbon was oxidized to CO_2_ to support the cellular demand for NADH. The data suggest that Xfp alone does not improve CCE efficiency for biomass growth. Finally, it is predicted that cells should not be able to grow on methane without an EMP or EDD pathway, as in that case there is no step that could convert acetyl-CoA into a C_3_ unit.

The genomic analyses predicted that a partial serine cycle might also contribute to carbon conversion to acetyl-CoA. A flux via the serine cycle was observed in in silico experiments with **Δ***pdh* and **Δ***xfp* knock-outs (Fig. 3d).

### Methane assimilation: nitrogen source

*M. buryatense* 5G(B1) is able to use nitrate, ammonia, and urea as sole sources of nitrogen [23, 24]. Genes encoding related enzymes and transporters were identified in the genome. Furthermore, genome-mining data suggest that the strain should be capable of nitrogen fixation. The predicted impacts of different nitrogen sources on biomass yields are shown in Table 2. As could be expected, ammonia and urea are the best sources for overall conversion. The strain is capable of ammonia utilization only at low pH (7.2–7.6); however, the cells grew slowly (Td = 12 h). We found that addition of sodium carbonate (0.1 g/L) can improve growth rate (Td = 5 h). Urea could be used at both high and low pH, and no significant differences in growth rate were observed. Urea seems to be an attractive alternative for nitrate at high pH methane conversion. Activation of the nitrogen fixation machinery is predicted to lead to biomass yield reduction; however, it might provide a more efficient nitrogen source than nitrate at high pH.

### Methane assimilation: sulfur source

*M. buryatense* 5G can use sulfate and sulfide as a source of sulfur [23, 24]. The FBA data predict that the substitution of sulfate with sulfite brings a mild increase in overall biomass yield. That is a very attractive feature of the strain, as H_2_S is the most common contaminant of biogas and natural gas, and thus could reduce the need for sulfate addition. Furthermore, the substitution of sulfate with sulfite is predicted to result in a mild increase in overall biomass yield.

## Conclusion

The results support the scenario in which methanol oxidation provides electrons for methane oxidation, with only up to 20 % of methane oxidation being driven by the electrons derived from NADH. The exact arrangement of the coupling (*direct* or *uphill*) remains to be established. While *direct coupling* is the most likely mode of methane oxidation, uphill transfer cannot be ruled out as an alternative at this time. Additional mutagenesis, enzymatic and proteomics studies are currently being conducted to establish the exact organization of initial steps of methane oxidation.

The metabolic models presented can provide effective tools for assessing the central metabolism of *M. buryatense* strain 5GB1. The model can be used to predict the impact of cultivation changes (nutrients, growth conditions) and genetic modifications (gene deletion or addition of novel genetic elements) on the performance of the biological system (methane consumption, growth rate, and phenotype), allowing screening prior to testing the most promising scenarios experimentally.

The metabolic model presented here is the first published genome-scale metabolic model of a methane-utilizing microbe and could also be used as part of a complex community modeling for assessing the methane cycle in a variety of ecosystems.

## Methods

### Biomass composition measurements

Cells of *M. buryatense* strain 5GB1 were grown to mid-exponential phase (OD_600_ 0.29 ± 0.01) using a mineral salts medium [25] in sealed vials or jars (50 or 250 mL culture in 250 mL vials or 1 l jars, supplemented with 50 or 250 mL of CH_4_, respectively) with shaking at 200 rpm at 28 °C. The optical density of cell cultures was measured on a Jenway 6320D spectrophotometer in plastic 1.5 mL cuvettes with a 1 cm path length. Cell samples were collected by centrifugation at 1500 g for 15 min. In bioreactor cultures, a premixed gas feed containing 5 % O2, 10 % CH4, 85 % N2 was continuously supplied to create fedbatch unrestricted growth conditions on methane (up to OD ≈ 1.5) [27].

Each parameter shown in Table 1 represents an average of measurements obtained from at least 2–6 biological replicates. Purification of DNA and RNA was performed using TRI Reagent (Sigma-Aldrich) according to the manufacturer’s instructions with the following modification: cells were homogenized with 0.2 g 1 µm silica beads (BioSpec) on a Bullet blender homogenizer (Next Advance) for 3 min. Cell biomass was lyophilized using a FreeZone (Labconco) lyophilizer and dry cell samples (two biological replicates, 1 g CDW each) were submitted to AminoAcids (https://www.aminoacids.com) for free and total protein-bound amino acid analyses and to Matrix Genetics (matrixgenetics.com) for fatty acid analyses. The concentrations of cellular LPS (5 biological replicates, each represented by a serial dilution of 1 mL culture collected from mid-exponential phase) were measured using a Pyrogent Plus test (Lonza) and Pierce™ LAL Chromogenic Endotoxin Quantitation Kit (Life Technologies, CA, USA). The concentration of extracellular polysaccharides was measured using a UV spectrophotometry with sulfuric acid assay [59]. For EPS estimations the supernatant samples (5–6 biological replicates, 25 mL each) were prepared as following: cells were grown till early mid-exponential phase and removed by centrifugation (1500*g* for 15 min, at 4 °C) and subsequent filtration through 0.11um PES filters (VWR, Radnor PA). In addition, a detailed analysis of exopolysaccharides (EPS) was carried out after freeze-drying the large molecular weight fraction after dialysis (3500 Da cut-off membrane) fraction of the culture supernatant. The dried material (2 mg) was subjected to a 4 M trifluoroacetic acid (TFA) hydrolysis, 4 h 100 °C. The monosaccharide composition of the hydrolyzed material was determined after TMS derivatization using an optimized procedure; in brief, the hydrolyzed TFA solution was evaporated under nitrogen to remove the residual acid, after which the resulting monosaccharides were derivatized with BSTFA (30 μL BSTFA + TMCS) at 60 °C for 120 min, followed by addition of 200 μl acetonitrile [NREL unpublished procedure], after which the silylated monosaccharides were identified by GC–MS (Agilent GC 7890A—Agilent MS 597C inert XL HSD) with the following analysis parameters: HP5 MS column (30 m × 0.25 mm i.d., 0.25 µm film thickness, Agilent), 1 µl injected, inlet at 280 °C, running at constant He flow of 1 mL/min. The oven ramp program (total of 71 min) was developed for optimal resolution of the anomeric silylated monosaccharide derivatives and can be summarized as follows: 80 °C for 2 min; 5 °C/min to 175 °C, hold for 1 min; 70 °C/min to 130 °C; 70 °C/min to 190 °C; 70 °C/min to 165 °C, hold for 1 min; 3 °C/min to 174 °C, hold for 2 min; 5 °C/min to 230 °C, hold for 3 min. Integration and calibration calculations were carried out by Agilent Chemstation for GC-FID (B.04.01).

*Sterols and squalene* were quantified in extractable lipids; in brief, chloroform:methanol (2:1 v/v) was used for lipid extraction from 100 mg of freeze-dried harvested cells using an accelerated solvent extractor (ThermoScientific), after which the lipids were analyzed by GC directly without derivatization. Identification was carried out by GC–MS (as above) with the following parameters: inlet at 350 °C, running at constant He flow of 1 mL/min. The oven ramp program (total of 29.75 min) was developed for optimal resolution of hydrocarbons, sterols and hopanoids; 100 °C for 1 min; 10 °C/min to 270 °C, hold for 7 min; 20 °C/min to 325 °C. Integration and calibration calculations were carried out by Agilent Chemstation for GC-FID after data was normalized for the addition of cholestane as an internal standard.

### Genome-scale metabolic network reconstruction

The scaffolds and annotations for 5GB1 were obtained from JGI’s IMG and converted into PathwayTools Pathologic input [60]. This included a list of all the genetic elements, the coordinates of predicted gene products, their annotations, including EC and GO terms where available, and sequences. Pathologic was then used to generate a purely computationally generated pathway/genome database. From there, the PathwayTools database was manually refined.

### RNA-seq data analysis

Samples of RNA were extracted from exponentially grown batch cultures (at OD = 0.6–0.7), grown as described above, and from cells grown in bioreactors (unlimited growth, OD = 1.5 [27]). The cellular activities were terminated by addition of *stop solution* [41], the cells (25 or 50 mL of culture) were collected by centrifugation at 4300×*g* at 4 °C for 10 min. Total RNA samples from two biological replicates of exponentially grown batch cultures and two biological replicates of bioreactor cultures were prepared and sequenced as previously described [41]. The raw reads from the sequencing run were aligned against the reference genome using *bwa* version 0.7.8-r455 under default options [61]. The alignments were post-processed using *SAMTools* version 0.1.19-44428 cd and assigned to ORFs using *HTSeq* version 0.5.4p3 in the *intersection*-*nonempty* mode and the final abundances were examined as *DeSeq* *2* normalized counts and reads per kilobase of transcript per million reads mapped (RPKM) [62–65].

### Urea, ammonium and sulfide tests

To test different sources of nitrogen and/or sulfur sources, the following modifications of the growth medium [25] were made: KNO_3_ (1 g/L) was replaced with 0.3 g/L of urea or 0.53 g/L of ammonium chloride; MgSO_4_ (0.2 g/L) was replaced with 0.2 g/L of sodium sulfide nonahydrate and 0.16 g/L of magnesium chloride. For low pH medium the carbonate buffer was omitted and phosphate solution (pH 7.2, final concentration in the growth medium 0.272 g/L KH_2_PO_4_ and 0.717 g/L Na_2_HPO_4_·12 H_2_O) was used as a buffer.

## Additional files

10.1186/s12934-015-0377-3 List of iMb5G(B1) model enzymatic reactions.
10.1186/s12934-015-0377-3 Gene expression profile in methane grown cells of *M. buryatense* 5GB1.
10.1186/s12934-015-0377-3 List of iMb5G(B1) transport reactions.
